# Indications for primary aldosteronism screening in people with hypertension and hyperparathyroidism: a multi-centre cohort study

**DOI:** 10.1007/s12020-025-04323-7

**Published:** 2025-07-03

**Authors:** A Jones, J. Tan, T. Dao, J. Tan, P. Wong, S. Sztal-Mazer, F. Milat, J. Yang, C. Gilfillan

**Affiliations:** 1 Department of Endocrinology and Diabetes, Alfred Health, Melbourne, VIC Australia; 2 Department of Endocrinology, Monash Health, Clayton, VIC Australia; 3 Eastern Clinical Research Unit, Eastern Health Clinical School, Monash University, Melbourne, VIC Australia; 4 Hudson Institute of Medical Research, Clayton, VIC Australia; 5 School of Public Health and Preventative Medicine, Monash University, Melbourne, VIC Australia; 6 Department of Medicine, School of Clinical Sciences, Monash University, Clayton, VIC Australia

**Keywords:** Primary aldosteronism, Hyperparathyroidism, Screening, Blood pressure, Calcium

## Abstract

**Purpose:**

Parathyroid hormone (PTH) excess is associated with hypertension while elevated PTH has been observed in primary aldosteronism (PA). This study aimed to determine the proportion of patients with hyperparathyroidism who met Endocrine Society criteria for PA screening, and to assess current screening practices.

**Methods:**

Multi-centre retrospective cohort study including patients attending outpatient endocrine clinics at three tertiary health services in Victoria, Australia between 2015–2019. Patients were included if they had an elevated PTH level and excluded if they had a secondary cause of hyperparathyroidism or a prior diagnosis of PA. Demographic, clinical and biochemical data were extracted from electronic medical records.

**Results:**

Of 275 patients with hyperparathyroidism, hypertension was present in 51.6%; including 62.4% of patients with hypercalcaemia and 35.5% of those with normocalcaemia. Overall,15.6% (43/275) had a guideline indication for PA screening, including 21.8% (36/165) of those with hypercalcaemia and 6.4% (7/110) of those with normocalcaemia. Of those with hypertension, 30% (43/142) had a guideline indication for PA screening. The most common indication for screening was hypertension and hypokalaemia (16/43). Despite this, only 9.3% (4/43) were screened, with one confirmed PA diagnosis.

**Conclusion:**

Hypertension is common in patients with hyperparathyroidism. A third of patients with hyperparathyroidism and hypertension had a guideline indication for PA screening, however screening remains substantially under-utilised. A prospective study is needed to evaluate the prevalence and impact of PA in patients with hyperparathyroidism.

## Introduction

Primary aldosteronism (PA) is an adrenal disorder characterized by excessive aldosterone production despite suppressed plasma renin [1]. Also known as Conn’s syndrome, PA was once thought to be a rare condition associated with severe hypertension and hypokalaemia. It is now recognized as the most common endocrine cause of secondary hypertension and can be found in patients with mild hypertension and normokalaemia [2, 3]. Patients with PA have an increased risk of cardiovascular events and stroke [4], renal disease, metabolic syndrome and an overall reduced quality of life [5]. Early identification of PA permits effective targeted treatment or potential surgical cure, yet PA remains largely underdiagnosed with <1% of eligible patients ever screened for the disease [6].

Several studies have demonstrated that patients with PA may have elevated parathyroid hormone (PTH) levels [7], which can be normalized by targeted PA treatment [8]. Studies have also shown that patients with PA are at a higher risk of osteopenia and osteoporosis compared to patients with essential hypertension, with a higher prevalence of vertebral fractures [9]. These phenomenona may be related to PTH excess, which has well-recognized effects on bone and calcium metabolism [10]. It is postulated that aldosterone may induce PTH secretion by increasing renal loss of calcium with subsequent decline in bone mineral density (BMD) [11]. The overall clinical pattern may therefore resemble normocalcaemic hyperparathyroidism, which is described as persistently high PTH levels with normal total and ionised calcium in the absence of known causes of secondary hyperparathyroidism [12]. The prevalence of normocalcaemic hyperparathyroidism ranges from 0.4–8.9%, dependent on selected populations and the exclusion criteria for secondary causes of hyperparathyroidism [13]. Currently, it is not standard practice to screen for PA in patients with normocalcaemic hyperparathyroidism [13].

In contrast to secondary hyperparathyroidism observed in patients with PA, primary hyperparathyroidism is characterized by autonomous secretion of PTH due to parathyroid hyperplasia or adenoma, resulting in hypercalcaemia [14]. Primary hyperparathyroidism is associated with hypertension in 40–60% of patients although the exact mechanism is unclear [15] and PA screening is not currently recommended. Importantly, untreated hyperparathyroidism is associated with increased cardiovascular mortality [16] which is partly attributed to concomitant hypertension observed in 40–60% of patients with primary hyperparathyroidism [17, 18]. It is the higher PTH levels, instead of the calcium levels, however, that correlates with higher all-cause and cardiovascular morbidity and mortality in untreated primary hyperparathyroidism [12].

Given the close association between PA and hyperparathyroidism, and the lack of data on patterns of PA screening amongst patients with hyperparathyroidism, we sought to examine the proportion of patients with hyperparathyroidism who had a guideline indication for PA testing, comparing those with normal calcium levels versus hypercalcaemia. We hypothesised that PA may be an under-recognised cause of elevated PTH, and there would be more patients with indications for PA testing in the normocalcaemic group than the hypercalcaemic group.

## Materials and methods

### Study design, setting and participants

We conducted a multi-centre retrospective cohort study at three tertiary health services in Victoria, Australia. Study participants included adults (18 years and above) who attended outpatient endocrinology and bone health clinics at the study sites between a five-year period (2015–2019, prior to COVID-related restrictions to in-person consultations) and had an elevated PTH concentration in the pathology database within each health service. Patients diagnosed with secondary causes of hyperparathyroidism (vitamin D < 50 nmol/L, estimated glomerular filtration rate [eGFR]<60 mL/min/1.73 m^2^, or other secondary causes reported by the clinician including familial hypocalciuric hypocalcaemia [FHH], renal or bone disease), were excluded as well as patients with known primary aldosteronism. Treatments for hyperparathyroidism included parathyroidectomy, cinacalcet, bisphosphonate, hydration and monitoring. Indications for PA screening were defined as per the Endocrine Society Criteria [19]. According to this guideline, screening with an aldosterone-to-renin ratio is recommended if any of the following eight criteria are met.; sustained blood pressure >150/100 in three separate measurements taken on different days, hypertension (>140/90 mmHg), resistance to three conventional anti-hypertensive drugs, hypertension controlled with four or more medications, hypertension and spontaneous or diuretic-induced hypokalaemia, hypertension and adrenal incidentaloma, hypertension and sleep apnoea, hypertension and a family history of early onset hypertension or a cerebrovascular event <40 years, or all hypertensive first-degree relatives of patients with PA [19]. Aldosterone renin screening was undertaken following withdrawal of confounding medications for at least 4 weeks [19].

The Human Research Ethics Committees of the three health services approved this study (Alfred Health Project Number 450/21, Eastern Health QA21-045, Monash Health RES-21-0000-440Q – 77423). Consents from participants were not required by the ethics committees as data were collected retrospectively and fully de-identified.

### Data collection and storage

Data were extracted from electronic medical records, including demographics, patients’ characteristics, and pathology results. Demographic data included sex, age, and country of birth. Patients’ characteristics included body mass index, blood pressure (BP) values, antihypertensive medications, comorbidities, family history, and treatment received for hyperparathyroidism. BP measurements were collected within the period of 12 months prior to and after the time point with the highest PTH level. Three sets of BP values were collected where available and were averaged to determine the indication for PA testing. The number and classes of antihypertensive medications taken were recorded. The comorbidities recorded were past hypokalaemia, obstructive sleep apnoea, chronic kidney disease, adrenal adenoma, and osteoporosis. Hypokalaemia was defined as any potassium level below the reference range or the use of potassium supplements, between first clinic presentation and post-hyperparathyroidism treatment presentation. The presence of obstructive sleep apnoea, chronic kidney disease, adrenal adenoma, and osteoporosis was recorded if the diagnosis had been established. If there was insufficient documentation/imaging to determine the presence of a condition, it was classified as unknown. Chronic kidney disease was classified by stages depending on eGFR results. Pathology results included serum corrected PTH, vitamin D, calcium, potassium, ionised calcium, phosphate, albumin, creatinine, and urine calcium to creatinine ratio. These were collected at the initial visit, at the time of the highest PTH level, and at the first visit post-treatment. Family history of hypertension and/or stroke below age 40, or family history of PA were documented. The type of treatment for hyperparathyroidism was also collected. Indications for PA screening were recorded according to the Endocrine Society guideline [19]. In the event that the participant had multiple indications for PA screening, each indication was documented.

### Bias, study size, and quantitative variables

Selection bias was mitigated by including patients objectively based on their laboratory-specific PTH levels. Confounders were also minimised by excluding patients with secondary causes of hyperparathyroidism which may have different impacts on BP. The number of cases detected at the health services during the study period determined the sample size. Patients with serum calcium within the reference range at initial presentation were considered to have normocalcaemic hyperparathyroidism. A serum calcium level above the reference range indicated hypercalcaemic hyperparathyroidism. Patients were analysed according to these normocalcaemic and hypercalcaemic categories.

### Laboratory assays

Hyperparathyroidism was defined as a PTH concentration above the laboratory-specific reference range (Alfred Health 1.6–6.0 pmol/L, Eastern Health 1.6–6.9 pmol/L, Monash Health 1.5–7.0 pmol/L). The reference ranges for the corrected calcium concentration were 2.00–2.50 mmol/L in 2015 or 2.10–2.60 mmol/L in 2016–2020 at Alfred Health and 2.10–2.60 mmol/L at Eastern Health and Monash Health. Hypokalaemia was defined as a serum potassium <3.5 mmol/L for all services.

### Data analysis

Continuous variables were expressed as mean and sample size (n). Categorical variables were described as sample size (n) and proportion (%). The prevalence of hypertension and qualification for PA screening were calculated for each hyperparathyroidism category (n, %). The associations between PTH, calcium, potassium and blood pressure in each hyperparathyroidism category were analysed using ANOVA test. Linear modelling assessed the relationship between age, PTH, BMI and systolic blood pressure. Two-sided *p* < 0.05 were considered statistically significant. All statistical analyses were performed using IBM® SPSS® Statistics software version 27.01- 2023 (IBM Corporation, Armonk, NY, USA).

## Results

### Participant characteristics

**Fig. 1:**
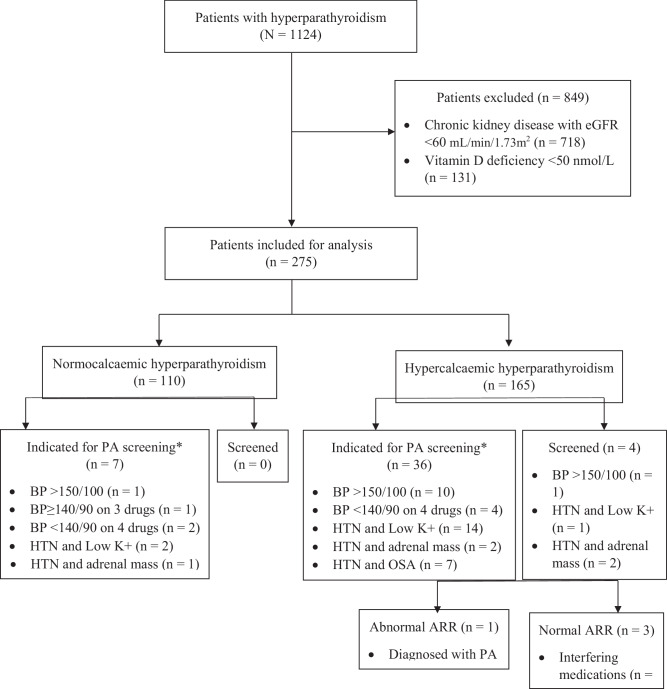
Screening for primary aldosteronism between hypercalcaemic and normocalcaemic groups in patients with hyperparathyroidism. PA primary aldosteronism, BP averaged blood pressure from three separate collections, HTN hypertension, low K+ = Hypokalaemia, OSA obstructive sleep apnoea. * Indication for PA screening was according to the Endocrinology Society guideline

A total of 1124 patients with hyperparathyroidism were identified at the 3 sites however 849 patients were excluded due to chronic kidney disease with eGFR <60 mL/min/1.73 m^2^ (*N* = 718) or vitamin D insufficiency/deficiency (<50 nmol/L (*N* = 131) (Fig. 1). No patients were excluded due to pre-existing PA. Of the 275 patients included, 110 patients had normocalcaemic hyperparathyroidism and 165 had hypercalcaemic hyperparathyroidism. Two hundred and six (74.9%) patients were female with a mean age of 70.4 years (SD 14.6). The baseline characteristics of participants in the normocalcaemic and hypercalcaemic groups and their comorbidities are described in Table 1.

**Table 1:** Baseline characteristics of participants

	Normocalcaemic	Hypercalcaemic	*P* Value
	(n = 110)	(n = 165)	
Age years (mean) (n)	68.3 (109)	71.8 (165)	0.99
Sex			
- Male (n, %)	26 (23.6%)	43 (26.2%)	0.81
- Female (n, %)	84 (76.4%)	122 (73.9%)	0.68
Body Mass Index (mean, kg/M^2^) (n)	26.6 (87)	29.1 (127)	0.39
Systolic Blood Pressure (mean, mmHg) (n)	128 (100)	132 (154)	0.62
Diastolic Blood Pressure (mean, mmHg) (n)	73 (101)	75 (164)	0.082
Hypertension (n, %)	39 (35.5%)	103 (62.4%)	<0.001
Hypokalaemia (n, %)	14 (12.7%)	28 (17.0%)	0.40
Hypertension and hypokalaemia	5 (4.5%)	20 (12.1%)	0.850
Obstructive Sleep Apnoea (n, %)	5 (4.5%)	11 (6.7%)	0.60
Chronic Kidney Disease (n, %)			
- Stage 1	2 (1.8%)	38 (23.0%)	<0.001
- Stage 2	47 (42.7%)	115 (69.7%)	<0.001
Adrenal Adenoma (n, %)	1 (0.9%)	2 (1.2%)	N/A
- Unknown	14 (12.7%)	5 (3.0%)	
Osteoporosis (n, %)	88 (80.0%)	101 (61.2%)	0.12
Calcium (mmol/L) (n)	2.4 (108)	2.8 (165)	<0.001
PTH (nmol/L) (n)	9.8 (109)	13.3 (165)	<0.001
Vit D (nmol/L) (n)	83 (100)	67 (162)	0.52
Potassium (mmol/L) (n)	3.8 (108)	3.9 (164)	0.084
DEXA score Lumbar Spine (mean, T-score) (n)	−1.92 (99)	−1.21 (126)	0.96
DEXA score Femoral Neck (mean, T-score) (n)	−2.16 (97)	−1.72 (124)	0.98

Where *n* number of patients, or expressed as a percentage of patients

In the hypercalcaemic group, 99.4% (164/165) had primary hyperparathyroidism, while one was indeterminate. Of 90 surgically managed patients in the hypercalcaemic group, 27 had blood pressure data pre- and post-surgery. In the normocalcaemic group, the cause of elevated PTH was not identified in the majority of patients (108/110, 98.2%) as patients with known secondary causes had been excluded from this study; two patients had parathyroid adenomas localised on sestamibi scans.

### Blood pressure in hyperparathyroidism

Of all the patients, 142/275 (51.6%) had a history of hypertension or elevated blood pressure (average of two to three measurements) in clinic. Hypertension was prevalent in 62.4% of the hypercalcaemic group and 35.5% of the normocalcaemic group (*p* < 0.001). Linear modelling demonstrated that BMI (*p* = 0.005), creatinine (*p* = 0.003) and age (*p* < 0.001) were associated with systolic blood pressure, while PTH had no effect (*p* = 0.693). There was no difference in BMI and age between the hypercalcaemic and normocalcaemic groups. Antihypertensive use was common in the whole group; 61 patients had one antihypertensive agent, 49 patients had two agents, 20 patients had three agents and 8 patients used four different agents. The proportion of patients on three or more anti-hypertensive medications was 18.4% in the hypercalcaemic group (19/103) and 23% in the normocalcaemic group (9/39). The proportion of patients using each class of anti-hypertensive agents did not differ significantly between the normocalcaemic and hypercalcaemic groups.

### Guideline indications for PA testing and outcomes of testing

A total of 43 patients (15.6% of overall cohort, 30.2% of those with hypertension) met the Endocrine Society guideline criteria for PA screening; 36 (21.8% of 165) in the hypercalcaemic group and 7 (6.4% of 110) in the normocalcaemic group (Table 2). Four patients (1.4% of the overall cohort, 2.8% of those with hypertension) were screened; two had hypertension and adrenal mass, one had hypertension and hypokalaemia and one had resistant hypertension. Of these four, one had a grossly abnormal ARR and was subsequently diagnosed with PA. Three patients had normal ARR but two of them were taking interfering medications which may have caused a false negative result.

**Table 2:** Patients qualifying for screening for primary aldosteronism as per endocrine society criteria^52^

Indication for PA testing	Normocalcaemic Hyperparathyroidism (n = 7)	Hypercalcaemic Hyperparathyroidism (n = 37)
Blood Pressure >150/100 (n, %)	1 (14.3)	10 (27.0)
Blood Pressure ≥140/90 on 3 drugs (n, %)	1 (14.3)	0
Blood Pressure <140/90 on 4 drugs (n, %)	2 (28.6)	4 (10.8)
Hypertension and Low Potassium (n,%)	2 (28.6)	14 (37.8)
Hypertension and Adrenal Mass (n,%)	1 (14.3)	2 (5.4)
Hypertension and Obstructive Sleep Apnoea (n,%)	0	7 (18.9)
Hypertension and Family History of Early Stroke (n,%)	0	0
Hypertension and First Degree Relative with PA (n,%)	0	0

In patients with hypertension, there was no statistically significant difference in the proportion with an indication for PA screening among those with hypercalcaemia or normocalcaemia (34.6%, 36/103 vs 17.9%, 7/39; *p* = 0.28). The most common indication for PA screening was a history of hypertension and hypokalaemia (16 patients; 14 in the hypercalcaemic group and 2 in the normocalcemic group), followed by blood pressure of >150/100 mmHg (11 patients) (Table 2). In hypercalcaemic patients who had an indication for PA screening and had surgical treatment of their primary hyperparathyroidism, PTH normalised in 85% (21/25) and diastolic, but not systolic blood pressure, was significantly reduced (80.3 mmHg to 72.2 mmHg, *p* = 0.038). Of the four patients with persistently elevated PTH, three had normalised calcium but all four had residual hypertension. They were not tested for PA and the cause of persistent PTH elevation was not found.

## Discussion

Our study of 275 patients with either normocalcemic or hypercalcaemic hyperparathyroidism across three tertiary centres found that hypertension was present in over 50% of the whole cohort; andifferent days, hypertension (>140/90 mmHgd 15.6% of the entire cohort had at least one guideline indication for PA screening. However, only 1.4% were screened for PA, which is consistent with current literature demonstrating low uptake of guideline recommendations for PA screening in Australia and internationally [20, 21].

The prevalence of hypertension in the normocalcaemic group (34.6%) was comparable to the baseline prevalence in this age group in Australia (34.2%) [22]. As expected it was higher in the hypercalcaemic hyperparathyroidism group (62.4%). We found that more hypercalcaemic patients had indications for PA screening than those with normocalcaemia. We had expected to find more patients with normocalcaemia to qualify for PA screening based on the hypothesis that hyperaldosteronism may be an under-diagnosed cause of secondary hyperparathyroidism. However, it is impossible to know which subgroup had the higher prevalence of PA without measuring plasma aldosterone and renin concentrations in all patients. To resolve this issue, prospective studies are needed to explore the outcomes of patients with hyperparathyroidism and hypertension who are formally investigated for PA.

We identified a high prevalence of hypokalaemia; around 15% of patients in the overall cohort, including 12.7% in the normocalcaemic group and 17% in the hypercalcaemic group. Whilst hypokalaemia has been reported in the setting of primary hyperparathyroidism due to associated renal tubular acidosis [23], it is unclear what can cause hypokalaemia in patients with normocalaemic hyperparathyroidism. Hypokalaemia and hypertension are typically observed in PA but only one of the patients with hypertension and hypokalemia was screened and subsequently diagnosed with PA in this study. Prospective evaluation of aldosterone and renin in affected patients will be insightful.

There is increasing evidence for an interaction between the renin-angiotensin-aldosterone system and calcium regulatory hormones [24, 25]. It is possible that aldosterone excess in patients with PA contributes to a rise in PTH without a concomitant increase in serum calcium due to aldosterone-mediated renal calcium loss [24]. How aldosterone excess may relate to hypercalcaemic hyperparathyroidism is less clear, but previous studies have demonstrated markedly decreased plasma aldosterone and renin levels [26] as well as decreased blood pressure [27] in patients with primary hyperparathyroidism after parathyroidectomy. On the other hand, animal studies have suggested that PTH may directly increase the secretion of aldosterone from the adrenal zona glomerulosa as well as indirectly by activation of the renin-angiotensin system [25]. A bidirectional relationship between hyperaldosteronism and hyperparathyroidism, with each condition perpetuating the other has thus been suggested [24, 28]. In our cohort, 15% of patients who underwent parathyroidectomy had persistently elevated PTH and systolic hypertension despite normalisation of calcium, raising the possibility of concurrent PA. In a large retrospective study of 462 patients with PA, PTH levels were higher in those with PA compared to “low renin without PA” and non-PA participants, despite comparable calcium levels and kidney function [29]. Normocalcaemic hyperparathyroidism was also more common in those participants with PA (38% vs 28%, *p* = 0.010) [29]. Large prospective studies investigating changes in PTH, aldosterone and renin following parathyroidectomy may help to further elucidate the interaction between these hormones.

A key limitation of our study is its retrospective nature, such that indications for PA screening could only be assessed on the basis of hospital notes and clinic blood pressure recordings. Data relating to some indications for PA testing, such as obstructive sleep apnoea, family history of hypertension, or the presence of an adrenal adenoma, may not have been available in hospital-based electronic records. Hence, the proportion of patients who had an indication for screening is likely an under-estimate of the true number who qualify for screening. Additionally, aldosterone and renin were not measured in most patients, precluding estimation of PA prevalence in the study cohort – a prospective study is required. With regard to hypercalcaemia, corrected calcium concentration was used to make the diagnosis as ionised calcium measurements were not available for the majority of patients. While we excluded patients with secondary hyperparathyroidism due to vitamin D deficiency and renal insufficiency, those on anti-resorptive therapy prior to PTH measurements were not excluded. The exclusion of patients with elevated PTH due to anti-resorptive therapy may further refine the cohort of patients with normocalcaemic hyperparathyroidism of unknown aetiology and improve identification of individuals who warrant PA screening.

Overall, we demonstrated that hypertension is common among patients with hyperparathyroidism and that a substantial proportion of these patients meet one or more criteria for primary aldosteronism (PA) screening. The mechanisms underlying hypertension in hyperparathyroidism, particularly in the setting of normocalcaemia, remain poorly understood, warranting further investigation into the potential role of PA. Consistent with previous reports from tertiary settings, including diabetes, nephrology, and emergency department cohorts, few eligible patients are ever screened for PA despite meeting guideline-recommended criteria. Our study, based on data from three large tertiary health services, provides further evidence that PA remains significantly under-recognised, even among patients with clear high-risk features such as concurrent hypertension and hypokalaemia.

Given the known prevalence of PA in 4–14% of patients with hypertension in primary care [3], a similar prevalence may be expected in patients with both hypertension and hyperparathyroidism. While our original hypothesis focused on normocalcaemic hyperparathyroidism as a potential context in which PA may be unmasked, our findings suggest that screening indications are similarly prevalent in both normocalcaemic and hypercalcaemic groups. A prospective study with formal biochemical evaluation for PA is needed before routine screening can be recommended in patients with normocalcaemic hyperparathyroidism in the absence of other established indications.

## Conclusion

A third of patients with hypertension and hyperparathyroidism have a guideline indication for PA testing. Whilst this suggests that PA screening should be considered in people with both hyperparathyroidism and hypertension, the role of routine testing in those with primary hyperparathyroidism is not clear because hypercalcaemia per se is associated with hypertension and thus PA screening in this group may not yield as many cases as it would in those without hypercalcaemia. Future prospective studies where patients are formally investigated for PA are required to appreciate the true prevalence of PA in hyperparathyroidism.

## Data Availability

The data that support the findings of this study are available from the corresponding author upon reasonable request.

## References

[1] D.A. Calhoun, M.K. Nishizaka, M.A. Zaman, R.B. Thakkar, P. Weissmann, Hyperaldosteronism among black and white subjects with resistant hypertension. Hypertension **40**(6), 892–896 (2002). 12468575 10.1161/01.hyp.0000040261.30455.b6

[2] S. Monticone, J. Burrello, D. Tizzani, C. Bertello, A. Viola, F. Buffolo, P. Mulatero, Prevalence and clinical manifestations of primary aldosteronism encountered in primary care practice. Journal Am. Coll. Cardiol. **69**(14), 1811–1820 (2017). 28385310 10.1016/j.jacc.2017.01.052

[3] R. Libianto, G.M. Russell, M. Stowasser, S.M. Gwini, P. Nuttall, J. Shen, J. Yang, Detecting primary aldosteronism in Australian primary care: a prospective study. Medical J. Aust. **216**(8), 408–412 (2022). 10.5694/mja2.51438 35218017

[4] S. Monticone, F. D’Ascenzo, C. Moretti, T.A. Williams, F. Veglio, F. Gaita, P. Mulatero, Cardiovascular events and target organ damage in primary aldosteronism compared with essential hypertension: a systematic review and meta-analysis. lancet Diabetes Endocrinol. **6**(1), 41–50 (2018). 29129575 10.1016/S2213-8587(17)30319-4

[5] G. Hanslik, H. Wallaschofski, A. Dietz, A. Riester, M. Reincke, B. Allolio, A. Hannemann, Increased prevalence of diabetes mellitus and the metabolic syndrome in patients with primary aldosteronism of the German Conn’s Registry. Eur J. Endocrinol. **173**(5), 665–675 (2015). 26311088 10.1530/EJE-15-0450

[6] Y.Y. Liu, J. King, G.A. Kline, R.S. Padwal, J.L. Pasieka, G. Chen, A.A. Leung, Outcomes of a specialized clinic on rates of investigation and treatment of primary aldosteronism. JAMA Surg. **156**(6), 541–549 (2021). 33787826 10.1001/jamasurg.2021.0254 PMC8014194

[7] C. Maniero, A. Fassina, T.M. Seccia, A. Toniato, M. Iacobone, M. Plebani, G.P. Rossi, Mild hyperparathyroidism: a novel surgically correctable feature of primary aldosteronism. Journal Hypertension **30**(2), 390–395 (2012). 10.1097/HJH.0b013e32834f0451 22179087

[8] E. Asbach, M. Bekeran, A. König, K. Lang, G. Hanslik, M. Treitl, M. Reincke, Primary and secondary hyperparathyroidism in patients with primary aldosteronism–findings from the German Conn’s registry. Experimental Clin. Endocrinol. Diabetes **128**(04), 246–254 (2020). 10.1055/a-1027-6472 31698477

[9] L. Petramala, L. Zinnamosca, A. Settevendemmie, C. Marinelli, M. Nardi, A. Concistrè, C. Letizia, Bone and mineral metabolism in patients with primary aldosteronism. International J. Endocrinol. **2014**, 836529 (2014). 10.1155/2014/836529 PMC4016829 24864141

[10] A.J. Koh, C.M. Novince, X. Li, T. Wang, R.S. Taichman, L.K. McCauley, An irradiation-altered bone marrow microenvironment impacts anabolic actions of PTH. Endocrinology **152**(12), 4525–4536 (2011). 22045660 10.1210/en.2011-1515 PMC3230047

[11] A. Vidal, Y. Sun, S.K. Bhattacharya, R.A. Ahokas, I.C. Gerling, K.T. Weber, Calcium paradox of aldosteronism and the role of the parathyroid glands. American J. Physiol.-Heart Circulatory Physiol. **290**(1), H286–H294 (2006). 10.1152/ajpheart.00535.2005 16373592

[12] G. Zavatta, B.L. Clarke, Normocalcemic hyperparathyroidism: a heterogeneous disorder often misdiagnosed?. JBMR **4**(8), e10391 (2020). 10.1002/jbm4.10391 PMC7422713 32803112

[13] F. Milat, S.K. Ramchand, M. Herath, J. Gundara, S. Harper, S. Farrell, C.M. Girgis, R. Clifton-Bligh, H.G. Schneider, S.M.C. De Sousa, A.J. Gill, J. Serpell, K. Taubman, J. Christie, R.W. Carroll, J.A. Miller, M. Grossmann, Primary hyperparathyroidism in adults-(Part I) assessment and medical management: Position statement of the endocrine society of Australia, the Australian & New Zealand endocrine surgeons, and the Australian & New Zealand bone and mineral society. Clinical Endocrinology. (2021). 10.1111/cen.14659 10.1111/cen.14659 34931708

[14] A.S. Salcuni, S. Palmieri, V. Carnevale, V. Morelli, C. Battista, V. Guarnieri, I. Chiodini, Bone involvement in aldosteronism. Journal Bone Miner. Res. **27**(10), 2217–2222 (2012). 22589146 10.1002/jbmr.1660

[15] J. Rastad, G. Akerström, S. Ljunghall, Mortality of untreated primary hyperparathyroidism-a nontraditional indication for parathyroid surgery?. American J. Med. **99**(5), 577–578 (1995). 10.1016/s0002-9343(99)80239-3 7485219

[16] J. Pepe, C. Cipriani, C. Sonato, O. Raimo, F. Biamonte, S. Minisola, Cardiovascular manifestations of primary hyperparathyroidism: a narrative review. European J. Endocrinol. **177**(6), R297–R308 (2017). 28864535 10.1530/EJE-17-0485

[17] N. Yu, G.P. Leese, P.T. Donnan, What predicts adverse outcomes in untreated primary hyperparathyroidism? the parathyroid epidemiology and audit research study (PEARS). Clinical Endocrinol. **79**(1), 27–34 (2013). 10.1111/cen.12206 23506565

[18] N.E. Cusano, S.J. Silverberg, J.P. Bilezikian, Normocalcemic primary hyperparathyroidism. Journal Clin. Densitom. **16**(1), 33–39 (2013). 10.1016/j.jocd.2012.12.001 PMC3564219 23374739

[19] J.W. Funder, R.M. Carey, F. Mantero, M.H. Murad, M. Reincke, H. Shibata, W.F. Young Jr, The management of primary aldosteronism: case detection, diagnosis, and treatment: an endocrine society clinical practice guideline. Journal Clin. Endocrinol. Metab. **101**(5), 1889–1916 (2016). 26934393 10.1210/jc.2015-4061

[20] G.L. Hundemer, H. Imsirovic, A. Vaidya, N. Yozamp, Screening rates of primary aldosteronism among individuals with hypertension plus hypokalemia: a population-based retrospective cohort study. J Hypertension **79**, 178–186 (2022). 10.1161/HYPERTENSIONAHA.121.18118 PMC8664996 34657442

[21] Y.Y. Lim, J. Shen, P.J. Fuller, L. Yang, Current patterns of primary aldosteronism diagnosis: delayed and complicated. Australian J. Gen. Pract. **47**, 712–718 (2018). 31195777 10.31128/AJGP-05-18-4587

[22] Australian Bureau of Statistics. (2022). National Health Survey. ABS, https://www.abs.gov.au/statistics/health/health-conditions-and-risks/national-health-survey/latest-release. Accessed 12 May 2022.

[23] J. Muthukrishnan, K.V. Hari Kumar, R. Jha, S. Jha, K.D. Modi, Distal renal tubular acidosis due to primary hyperparathyroidism. Endocrine Pract. **14**(9), 1133–1136 (2008). 10.4158/EP.14.9.1133. 10.4158/EP.14.9.1133 19158053

[24] A. Tomaschitz, E. Ritz, B. Pieske, A. Fahrleitner-Pammer, K. Kienreich, J.H. Horina, C. Drechsler, W. März, M. Ofner, T.R. Pieber, S. Pilz, Aldosterone and parathyroid hormone: a precarious couple for cardiovascular disease. Cardiovascular Res. **94**(1), 10–19 (2012). 10.1093/cvr/cvs092. 10.1093/cvr/cvs092 22334595

[25] J.M. Brown, A. Vaidya, Interactions between adrenal-regulatory and calcium-regulatory hormones in human health. Current Opin. Endocrinol., Diabetes Obes. **21**(3), 193–201 (2014). 10.1097/MED.0000000000000062. 10.1097/MED.0000000000000062 PMC4123208 24694551

[26] I. Sotornik, J. Stribrna, J. Hronova, V. Kocandrle, V. Janata, P. Taborsky et al., Changes in plasma renin and aldosterone after parathyroidectomy in patients with hyperparathyroidism. Casopis Lékarů Ceských **132**, 45–49 (1993). 8453649

[27] L. Brunaud, A. Germain, R. Zarnegar, M. Rancier, S. Alrasheedi, C. Caillard et al., Serum aldosterone is correlated positively to parathyroid hormone (PTH) levels in patients with primary hyperparathyroidism. Surger **146**, 1035–1041 (2009). 10.1016/j.surg.2009.09.041 19958930

[28] A. Vaidya, J.M. Brown, J.S. Williams, The renin-angiotensin-aldosterone system and calcium-regulatory hormones. Journal Hum. Hypertension **29**(9), 515–521 (2015). 10.1038/jhh.2014.125. 10.1038/jhh.2014.125 PMC4769794 25631218

[29] A. Ooi, H. Khan, M. Akram, P.J. Fuller, F. Milat, J. Yang, R. Libianto, Changes in parathyroidism hormone across the spectrum of renin-independent aldosteronism. J. Clin. Endocrinol. Metab. dgaf151 (2025). 10.1210/clinem/dgaf151. 10.1210/clinem/dgaf151 40052768

